# Items

**Published:** 1879-05

**Authors:** 


### Items.

---

"CALIFORNIA'S LAST, BEST GIFTS."—It is well known to most of our readers that one Dr. Bundy, of California, has been foisting upon the market, with the aid of a certain journal devoted to bringing before the public the products of a manufacturing firm, numerous *new preparations* of non-officinal drugs indigenous to his own State, claiming for them remarkable properties. Some of these were introduced under their own botanical names; for others names were drawn from the Spanish vernacular or were coined for them in the same language. These have been widely advertised and favorably commented upon by various medical and pharmaceutical journals.

Among other journals, *The Philadelphia Druggist and Chemist*, having perpetrated a lot of buncome concerning them, Dr. Gibbons has thought best to correct its statements, and give a brief sketch of some of the plants, in a leading article in the *Pacific Medical and Surgical Journal* (October, 1878). We draw upon his paper for much of the following.

*Yerba Santa* ("Holy Herb"). Eriodyction glutinosum, a shrub growing along the coast range and its foot-hills; used extensively in domestic medicine by the Spanish population; considered of some value in pulmonary and throat affections. Has been used more or less by regular practitioners for years, although regarded as still on trial. *Not* introduced by Dr. Bundy.

*Berberis Aquifolium.*—One of four species of berberis or barberry found in California. In the secondary list of the U.S.P. The genus is very old, and its active principle *berberina* well known. B. pinnata is probably what Bundy uses.

*Cascara Sagrada* (Sp. for "Sacred Bark").—No such plant known to botanists. Dr. Gibbons received a specimen from a Cincinnati druggist which he was convinced was the article sold as cascara sagrada, which proved to be a species of rhamnus, or buckthorn, of which four species are found in California. The properties of rhamnus are well known, and here we have an explanation of the wonderful virtues of cascara sagrada in constipation and allied difficulties. Bundy claims to be the discoverer of this remedy, known and used three hundred years before he was born.

*Yerba Reuma.* Bryzopyrum Spicatum (Hooker).—A grass covering the beaches and marshes along the coast. Has slightly astringent properties.

*Grindelia* (Composital).—G. Robusta is a known species. G. Hirsutula has been used in rhus poisoning. G. Squarrosa was described in Torrey & Gray's botany, but in the new California flora of Dr. Gray was omitted because it was not a well-marked species. G. Robusta is probably of some value, but Bundy's cautions as to the necessity of noting the difference between the robusta and squarrosa are simply bosh.

As Dr. Gibbons says, "it is not agreeable to deal in personalities," but the truth should be known. The writer was informed months ago, by the representative of a well-known Eastern house, that this I. H. Bundy was an eclectic, not to say charlatan, and the article from which we quote, written by one who knows still more of him, only corroborates the statement. That such a man can boast of his pseudo "discoveries," of his tremendous labors connected therewith, and can dignify them with any such phrase as stands at the head of this article, is bad enough, but that his worthless writings should be given such exensive notice, and his statements be made to fall, *ex cathedra*, upon our ears, is intolerable, and we are glad to do our share of publishing the truth.

APOLLINARIS WATER.—Some of the New York papers, who, following the example of many of their most eminent European *confreres*, endorsed the dietetic value, purity and natural origin of the Apollinaris water, must have felt a little shaken in their judgment, or, at least, annoyed at the implied slur upon their accuracy by the publication and extensive advertisement of trade attacks upon the water, which alleged that the Apollinaris water sold here in bottles is not the water of the spring, and is not charged with its natural gas. The recent publication of certificates by Oscar Liebreich, Wauklyn, Herman Weber and others, re-affirming the absolute accuracy of the claims of the Appollinaris of commerce to its title of a natural mineral water, has had a re-assuring effect; but we think that the certificates now issued and just placed in our hands will, nevertheless be valuable as setting the matter entirely at rest. It would not be possible to mention four names of greater weight on such a subject, or more sure to receive entire assent to any statements which they make, than those of Prof. Hoffman, of Berlin; the illustrious Kekule; Frankland, the leading water analyst of Great Britian; Odling, the British chemical juror at our centennial exhibition and the professor of chemistry in the University of Oxford. All four have been induced to repair to the springs to examine every detail personally, to compare the water issuing from the spring with the water of commerce, and to investigate the process of bottling. Each for himself, and in his own words, elaborately reports the result of his personal investigation, and each arrives at the same conclusion, namely, that the Apollinaris natural mineral water of commerce is the natural mineral water of the Apollinaris spring, bottled at the spring, charged only with its own gas, and with the same proportion of the gas as exists in the water at the depth at which it is drawn from the spring. The agents of the U. S. Treasury Department in Europe had been instructed from Washington to institute for themselves an independent inquiry, so as to ascertain and place the facts beyond doubt. They have done so, and have officially reported likewise to the government, that there is no foundation whatever for the charges made, and that the Apollinaris water sold here is, beyond doubt, the water of the spring, bottled on the spot and charged with its own gas. Thus the New York doctors may rest satisfied that they are justified in their generation, and that *this* "great European novelty," at all events, is what it represents itself to be.

